# Obesity Leads to Tissue, but not Serum Vitamin A Deficiency

**DOI:** 10.1038/srep15893

**Published:** 2015-11-02

**Authors:** Steven E. Trasino, Xiao-Han Tang, Jose Jessurun, Lorraine J. Gudas

**Affiliations:** 1 Department of Pharmacology, Weill Cornell Medical College of Cornell University, New York, NY 10065, USA; 2 Department of Pathology, Weill Cornell Medical College of Cornell University, New York, NY 10065, USA.

## Abstract

Obesity negatively affects multiple metabolic pathways, but little is known about the impact of obesity on vitamin A (VA)[retinol (ROL)], a nutrient that regulates expression of genes in numerous pathways essential for human development and health. We demonstrate that obese mice, generated from a high fat diet (HFD) or by genetic mutations (i.e., *ob/ob*; *db/db*), have greatly reduced ROL levels in multiple organs, including liver, lungs, pancreas, and kidneys, even though their diets have adequate VA. However, obese mice exhibit elevated serum VA. Organs from obese mice show impaired VA transcriptional signaling, including reductions in retinoic acid receptor (RARα, RARβ2 and RARγ) mRNAs and lower intracellular ROL binding protein Crbp1 (RBP1) levels in VA-storing stellate cells. Reductions in organ VA signaling in obese mice correlate with increasing adiposity and fatty liver (steatosis), while with weight loss VA levels and signaling normalize. Consistent with our findings in obese mice, we show that increasing severity of fatty liver disease in humans correlates with reductions in hepatic VA, VA transcriptional signaling, and Crbp1 levels in VA storing stellate cells. Thus, obesity causes a “silent” VA deficiency marked by reductions in VA levels and signaling in multiple organs, but not detected by serum VA.

Vitamin A (VA), an essential micronutrient, refers to a family of compounds that includes all-trans retinol (ROL) and its metabolites, all-trans retinaldehyde (RAL), all-trans retinoic acid (RA), and retinyl-esters (RE)^1^. VA and VA signaling are required for normal embryonic development, and in adults for many cellular functions, including maintenance of epithelial integrity, vision, and immune functions^2^; VA metabolites are also used in the treatment of a number of cancers^3^,^4^. There are published data that reduced tissue VA levels and RA signaling in animals on a VA deficient diet lead to the development of some cancers^5^, altered immune functions^6^,^7^, and cognitive decline^8^. VA signaling can regulate pathways adversely affected in states of obesity, such as hepatic lipid metabolism^9^,^10^,^11^, pancreatic endocrine functions^12^,^13^, adipogenesis^14^,^15^, and immune functions^16^,^17^,^18^. In rodents, perturbations of VA signaling by dietary VA depletion or genetic manipulation of VA signaling pathways can promote adipose tissue expansion and obesity^19^,^20^,^21^, and dietary or pharmacological administration of VA or RA can reduce obesity and promote energy utilization in obese mice^22^,^23^,^24^. However, whether obesity alters VA status, VA-signaling, and consequently, the abilities of organs to function properly has not been previously addressed. Here, we demonstrate for the first time that even with adequate dietary VA, obesity dramatically reduces VA levels and signaling in major organs.

## Results and Discussion

### High Fat Diet and Genetically Obese Mice Exhibit Reductions in Vitamin A in Multiple Organs Despite Having Adequate Dietary Vitamin A

**Figure 1:**
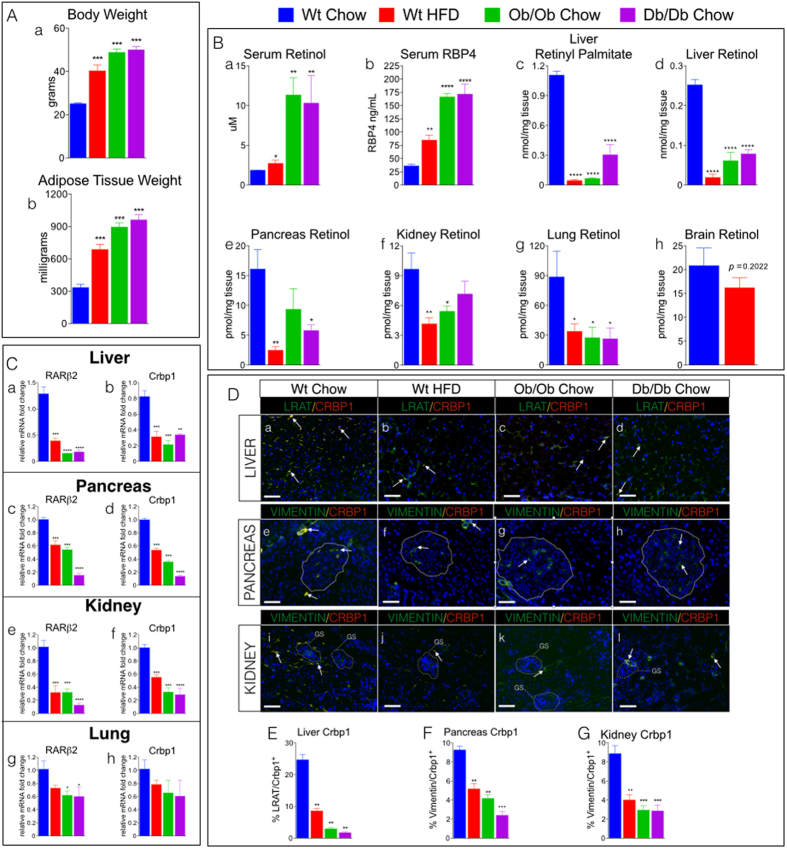
HF Diet and Genetic Models of Obesity Have Decreased Tissue Levels of Vitamin A. (**A**) (**a**) Body weights of (wt) C57/BL6 mice fed either a standard lab chow diet (chow) or high fat diet (HFD) for 4 months, and 12 week-old *ob/ob* mice and *db/db* mice fed a standard lab chow diet. (**b)** Adipose tissue (epididymal) weight in milligrams (mg) of wet weight from mice described in A. (**B) (a)** Serum levels of retinol (μM) and (**b)** serum retinol binding protein-4 (RBP4) (ng/ml) in mice described in A. Quantitation of high performance liquid chromatography (HPLC) of (**c)** liver retinyl-palmitate and (**d)** liver all-trans retinol (ROL) levels, and ROL levels in **(e)** pancreas, **(f)** kidney, **(g)** lung, and **(h)** brain from mice described in **A**. (**C)** Relative mRNA levels of retinoic acid receptor β2 (RARβ2) and cellular retinol binding protein-1 (Crbp1) in **(a,b)** liver, **(c,d)** pancreas, **(e,f)** kidneys, and **(g,h)** lungs from mice described in A. (**D)** Representative images of liver, pancreas and kidney double immunofluorescence stained with antibodies against Crbp1 (red) and the stellate cell (SC) marker LRAT (green) in **(a–d)** liver, and Crbp1 (red) and the SC marker Vimentin (green) in **(e–h)** pancreas and islets (white dotted lines) and **(i–l)** kidney (glomerulus (GS), white dotted lines) from mice described in A. (**D)(a–h)** Crbp1 positive SCs (yellow/orange) marked with white arrows. Magnification 200X, Scale Bars = 50 μm. (**E–G)** Quantitation of % percentage of Crbp1 positive stellate cells (SCs) in (**E)** liver, (**F)** Pancreas, and **(G)** Kidneys from mice described in A.

Adult wild type (wt) C57Bl/6 male mice fed a high fat for 4 months (HFD (45% fat/kcal); standard VA supplementation-see Methods) and 12-week-old, chow-fed *ob/ob* and *db/db* male mice^25^,^26^ showed increased body weights (BW) and adiposity compared to wt chow-fed mice (standard chow (13% fat/kcal)) (Fig. 1A, a,b). We determined the VA status of HFD-fed wt and *ob/ob* and *db/db* mice by measuring serum concentrations of all-trans retinol (ROL) and the major serum ROL carrier protein, retinol binding protein-4 (RBP4)^27^. Adult wt mice on a normal chow diet had serum ROL concentrations of 1.8 μM, (Fig. 1B), similar to the ROL serum concentrations in humans of normal weight^28^. Serum ROL and RBP4 in HFD-wt and in chow-fed *ob/ob*, and *db/db* mice were elevated by ~1.5–5 fold compared to levels in chow-fed wt (Fig. 1B)). The elevated serum ROL and RBP4 levels in *ob/ob* and *db/db* mice were striking and prompted us to ask if the serum ROL profiles of obese mice reflected their hepatic levels of ROL and retinyl-palmitate (RP), the major lipidated storage form of VA. Hepatic levels of these forms of VA are the benchmark for assessing VA status in humans^29^. We found that liver RP levels were reduced by 70–90% (Fig. 1B(c)) and ROL by 60–70% (Fig. 1B(d); Fig. S1 (A–D)) in HFD-fed wt and chow-fed *ob/ob* and *db/db* mice compared to chow-fed wt mice. We also measured ROL in several other organs and discovered that ROL levels in the pancreas, kidneys, and lungs of HFD-fed wt and chow-fed *ob/ob* and *db/db* mice were reduced by 60–70% (Fig. 1B(e–g), Fig. S1 (A–D)) compared to chow-fed wt. In contrast, ROL was not significantly reduced (~18%) in brain tissue from obese mice (Fig. 1B (h)), though there was a trend towards a small reduction. Vitamin A deficiency (VAD) in humans is defined as hepatic stores of VA below 0.07 nmoles of ROL per milligram tissue^29^,^30^. The mean hepatic levels of ROL and REs in HFD-wt, *ob/ob*, and *db/db* mice were 0.053 ± 0.017 (SEM) nmoles/mg of tissue (Fig. 1B (d)), placing the VA status of these obese mice below the clinical cutoff for VAD in adult humans^30^. However, whereas in VAD resulting from malnutrition *both* tissue and serum ROL levels are reduced^30^, serum levels of ROL were *elevated* in these obese mice despite the major reductions in hepatic and other organ VA levels (Fig. 1B (a)).

### Vitamin A Signaling is Greatly Reduced in Multiple Organs of Obese Mice

The major VA metabolite, RA, transcriptionally activates genes by acting as an agonist for all three retinoic acid receptors (RARα, β, γ)^1^,^2^. RA also increases mRNA transcripts of RARβ2 (gene ID: 218772)^31^, and the vitamin A binding protein cellular retinol-binding protein-1 (Crbp1 (Rbp1) gene ID: 19659)^32^. Crbp1 is required for intracellular delivery of ROL to acyltransferase enzymes such as lecithin:retinol acyltransferase (LRAT), which catalyzes the conversion of retinol to retinyl-esters (RE), and to enzymes which metabolize ROL to RA^33^,^34^,^35^. Crbp1 and RARβ2 are essential for normal VA metabolic homeostasis and signaling^31^,^34^, and as our laboratory has previously demonstrated that tissue mRNA levels of these genes are reliable molecular indicators of vitamin A signaling and responsiveness *in vivo*^12^, we investigated the consequences of the obesity-induced reductions in organ ROL levels on VA signaling by measuring the relative mRNA levels of RARβ2, Crbp1 and the other RAR isotypes, RARα (gene ID: 19401) and RARγ (gene ID: 19411), using real-time PCR (qRT-PCR). *RAR*α (Fig. S2(A,C,E,G)), *RARβ2* (Fig. 1C(a,c,e,g)), *RAR*γ (Fig S2.(B,D,F,H)) and *Crbp1* (Fig. 1C(b,d,f,h)) transcripts are reduced in liver, pancreas, kidneys, and lungs of HFD-fed wt and chow-fed genetically-obese mice compared to chow-fed wt. We also measured mRNA levels of retinoid X receptor (RXR) α (gene ID: 20181), and stimulated by retinoic acid 6 (Stra6) (gene ID: 20897), two genes also involved in vitamin A signaling and homeostasis^1^. *RXRα* transcript levels were unchanged in liver, pancreas, kidney, and lungs of HFD-fed wt and chow-fed genetically-obese mice compared to chow-fed wt mice (Fig. S2 (C,G,K,O)). *Stra6* mRNA levels were undetected in livers of all experimental mice (Fig. S2 D), and unchanged in pancreas, kidney, and lungs of HFD-fed wt and chow-fed genetically-obese mice compared to chow-fed wt mice (Fig. S2 (H,L,P)). The lower mRNA levels of *RAR*α, *RARβ2*, *RAR*γ, and *Crbp1* in the tissues of HFD-fed wt and chow-fed genetically-obese mice demonstrate that the reductions in tissue ROL levels in obese mice resulted in reductions in VA-mediated signal transduction in these organs.

### Vitamin A Storing Cells Have Reduced Expression of Cellular Retinol Binding Protein-1

Stellate cells (SCs) are the major VA storing cells both in liver and in a number of extra-hepatic tissues, including pancreas, kidneys, lungs, and intestine, and SCs express high levels of Crbp1^11^. Thus, we performed double immunofluorescence labeling using antibodies against Crbp1 and SC markers (LRAT in liver; vimentin in pancreas and kidney). Whereas most SCs from chow-fed wt mice express Crbp1 in liver, kidneys, and pancreas (Fig. 1D (a,e,i), double labeled; white arrows), fewer SCs from HFD-fed wt and chow-fed *ob/ob* and *db/db* mice expressed detectable Crbp1 (Fig. 1E,F,G & D(b–d,f–h,j–l)). The reductions in Crbp1 positive SCs from HFD-fed wt and chow-fed *ob/ob* and *db/db* mice are similar to the reductions we detected in adult mice with mild to severe dietary-induced VA deficiency (VAD)^12^.

Crbp1 is an important cellular ROL trafficking protein and is critical for VA signaling^34^. Adult mice that lack Crbp1 show a reduced capacity for hepatic uptake of dietary ROL and 50% reductions in SC RE levels in liver^36^. Crbp1 null adult mice also have marked reductions of ROL levels in lung, and ROL and RP levels in kidneys^36^, but do not have reductions in serum VA and do not show classic symptoms of VAD (e.g. impaired vision, low serum VA) unless fed a VA deficient diet for an extended period of time^36^. Given the overlap in the VA status of obese mice (Fig. 1B) and Crbp1 null mice^36^, coupled with the reductions in the percentages of Crbp1 positive SCs and VA signaling in obese mice (Fig. 1D,C), we conclude that obesity causes a biochemical and functional VA deficiency in tissues that is not reflected by serum ROL levels.

### Weight Loss Reverses Reductions in Tissue Vitamin A Levels and Signaling

**Figure 2:**
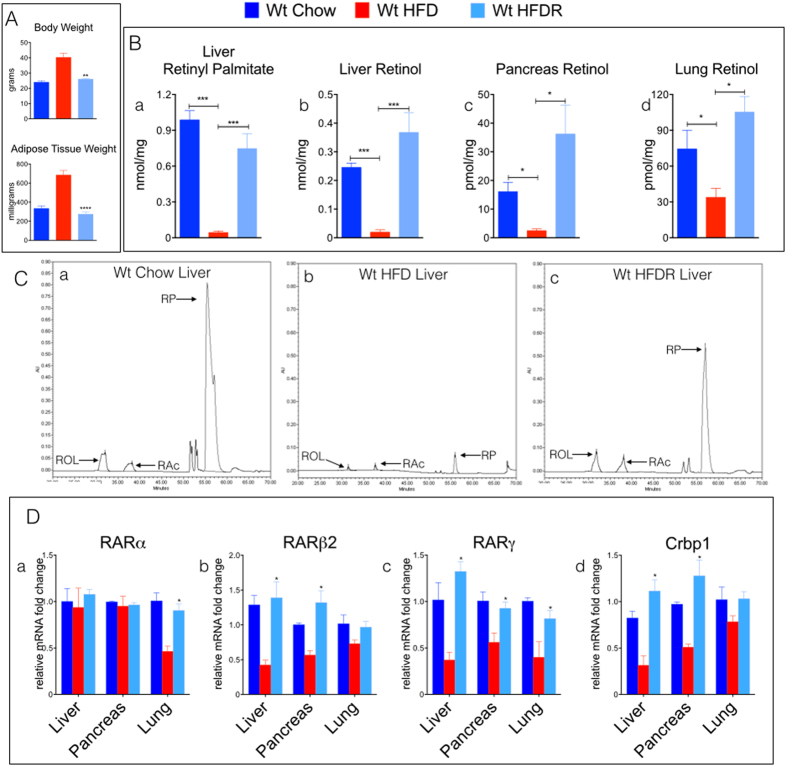
Weight Loss Restores Tissue Vitamin A Levels and Vitamin A Signaling in Obese Mice. **(A) (a)** Body weights of (wt) C57/BL6 mice fed either a standard lab chow diet (chow) or high fat diet (HFD) for 4 months, and wt mice that were previously obese and then switched back to a chow diet for 6 weeks (HFDR). (**b)** Adipose tissue (epididymal) weights in milligrams (mg) of wet weight from mice described in **(a)**. (**B)** Quantitation of high performance liquid chromatography (HPLC) of (**a)** liver retinyl-palmitate and all-trans retinol levels in **(b)** liver, **(c)** pancreas, and **(d)** lung from mice described in A. **(C)** Chromatographic tracings of VA (absorption units (AU)) extracted from livers of **(a)** wt mice fed a standard lab chow diet, **(b)** wt mice fed a HFD for 4 months, and **(c)** wt mice that were previously obese (HFD-fed for 4 months) and then switched back to a chow diet for 6 weeks (HFDR). All-trans retinol (ROL- arrow, ~32-minute retention time) and retinyl-palmitate (RP- arrow, ~55-minute retention time) were detected at a wavelength of 325 nm and identified by a match of retention times of pure retinoid standards. RAc-arrow, retinyl acetate, added control for extraction efficiency. **(D)** Relative mRNA levels of retinoic acid receptors **(a–c)**; RARα, RARβ2, RARγ respectively) and cellular retinol binding protein-1 (Crbp1) **(d)** in liver, pancreas, and lungs from mice described in A.

To address the question of whether the reductions in tissue ROL in obesity can be reversed by reductions in body weight, we conducted a “rescue” experiment in which wt mice fed a HFD for 4 months were then switched back to a standard chow diet for 6 weeks and re-tested for tissue ROL levels. Obese wt mice rescued with 6 weeks of chow-feeding (HFDR) lost weight and showed body weight and adiposity similar to age matched wt mice that had been fed chow for the entire 5 ½ months (Fig. 2A). The ROL and RP levels in liver (Fig. 2B(a,b)) and ROL levels in pancreas (Fig. 2B(c)) and lungs (Fig. 2B(d)) from HFDR-wt were also similar to those in the 5 ½ month chow-fed wt mice. The *RARα*, *RARβ2*, *RAR*γ, and *Crbp1* transcript levels were not reduced in livers, pancreata, and lungs of HFDR-wt compared to chow-fed wt mice (Fig. 2D(a–d)). These experiments show that: i) reductions in tissue ROL in obese states occur in parallel to increases in adipose tissue and body weight; ii) weight reduction in previously obese mice can restore tissue ROL levels and signaling, indicating that the VA tissue functional deficiencies are reversible; and iii) because declines in tissue ROL and signaling occurred in both chow-fed genetically obese (*ob/ob*, *db/db*) and HFD-fed wt mice, we suggest that obesity itself leads to this functional VAD in many organs.

**Figure 3:**
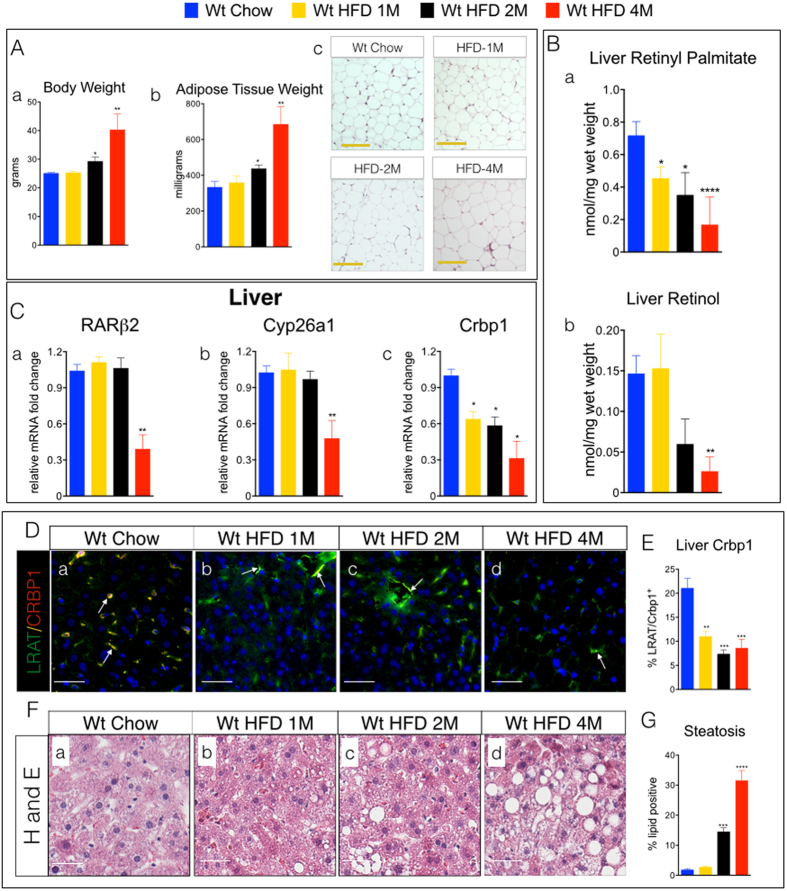
Liver Levels of Vitamin A and Cellular Retinol Binding Protein-1 are Reduced with Increasing Obesity. **(A) (a)** Body weights of (wt) C57/BL6 mice fed either a standard lab chow diet (chow) or high fat diet (HFD) for 1 month (1M), 2 months (2M) or 4 months (4M). (**b)** Adipose tissue (epididymal) weights in milligrams (mg) of wet weight from mice described in A. (**c)** Representative images of hematoxylin and eosin stained epididymal adipose tissue depots from mice described in (**a)**. Magnification 200X, Scale Bars = 50 μm. (**B)** Quantitation of **(a)** retinyl-palmitate and **(b)** all-trans retinol levels in livers from mice described in A using of high performance liquid chromatography (HPLC). (**C)** Relative mRNA levels of **(a)** retinoic acid receptor β2 (RARβ2), **(b)** cytochrome P450-member 26a1 (Cyp26a1), and **(c)** cellular retinol binding protein-1 (Crbp1), in livers from mice described in A. **(D) (a–d)** Representative images of liver double immunofluorescence stained with antibodies against Crbp1 (red) and LRAT (green) from mice described in A. Crbp1 positive SCs (yellow/orange) marked with white arrows. Magnification 200X, Scale Bars = 50 μm. **(E)** Quantitation of % percentage of Crbp1 positive stellate cells (SCs) from D. (**F) (a–d)** Representative images of hematoxylin and eosin (H & E) stained livers from mice described in A. Magnification 200X, Scale Bars = 50 μm. (**G)** Quantitation of % percentage hepatic steatosis (% hepatocytes with gross macro- or micro-vesicular lipid) in from mice described in A.

What remained unclear is whether reductions in tissue VA occurred in parallel to the onset of obesity or preceded it. We then conducted a time course experiment using wt mice fed a HFD for various time periods. HFD-fed wt were tested for tissue ROL levels after 1 month (1M), 2 months (2M), and 4 months (4M) on a HFD. Compared to chow-fed wt mice, body weights (BW), adipose tissue, and adipocyte size were not increased in 1M HFD (Fig. 3A(a–c)), but were in 2M and 4M HFD-wt mice (Fig. 3A(a–c)). Livers from 1M, 2M and 4M HFD-wt mice showed reductions in RP of ~31%, 52% and 76%, respectively (Fig. 3B(a)). ROL levels were not reduced in livers of 1M HFD-fed wt (Fig. 3B(b)), but were reduced by ~60% and ~80% in livers of 2M and 4M HFD-wt mice, respectively (Fig. 3B(b)). We also measured hepatic transcript levels of *RARβ2*, *Crbp1*, and another VA-regulated gene, *Cyp26a1*, and found that *RARβ2* and *Cyp26a1* transcripts were not reduced in livers of 1M and 2M HFD-fed mice, but were reduced in 4M HFD-fed mice (Fig. 3C(a,b)). In contrast, *Crbp1* mRNA levels were reduced in livers of HFD-fed mice beginning at 1M of the HFD (Fig. 3C(c)). We also performed immunofluorescence labeling studies to assess SC expression of Crbp1 and found that, consistent with the hepatic *Crbp1* mRNA levels from 1M and 2M HFD-wt mice, the percentages of Crbp1 positive SCs were also reduced in livers in 1M and 2M HFD-wt mice (Fig. 3D (a–d)). These data demonstrate that hepatic reductions in VA occur in parallel with increasing obesity and adiposity, but reductions in VA signaling do not occur (Fig. 3C(a,b)) until hepatic levels of both major forms of VA (ROL and RP) are reduced by >~70% (Fig. 3B(a,b)). Our previous studies demonstrated that reductions in pancreatic SC Crbp1 reflected pancreatic tissue VA status in VA deprived mice^12^. Thus, in these HFD experiments the decrease in hepatic SC Crbp1 mRNA and protein levels (Fig. 3C (c)) is one of the earliest molecular changes that occurs with increasing obesity.

### Steatosis is Associated with Reductions of Vitamin A Levels and Signaling in Human Livers

The reductions in hepatic VA and SC Crbp1 levels occur concomitantly with increases in hepatic steatosis in obese mice (Fig. 3F(a–d)), but without evidence of hepatitis (inflammation) or fibrosis (collagen deposition) measured by trichrome staining (Fig. S3A). A switch to normal chow rescued HFD-wt mice, which showed restoration of hepatic VA levels (Fig. 2B(a,b)), improved VA signaling (Fig. 2D(a–d)), and reductions in hepatic steatosis after weight loss (Fig. 2A) relative to HFD fed-wt mice (Fig. S3B).

**Figure 4:**
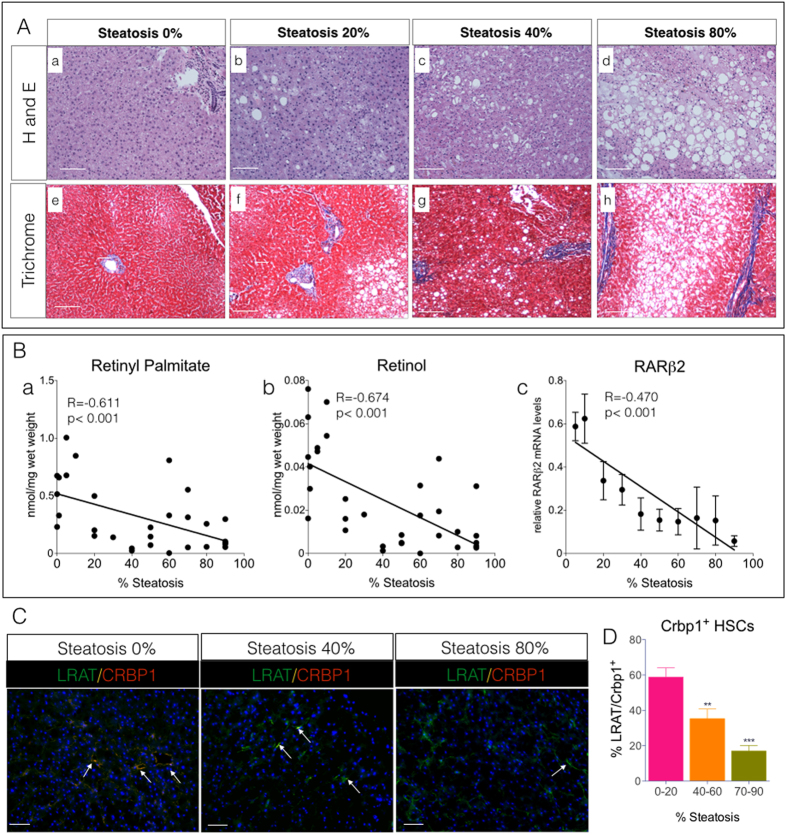
Vitamin A Levels and Signaling are Reduced in Human Livers with Steatosis. **(A)** Representative hematoxylin and eosin **(a–d),** and trichrome **(e–h)** stained human livers with increasing percentage of fatty liver (0–80% steatosis). Magnification 100X, Scale Bars = 25 μm. (**B)** Regression analysis of hepatic **(a)** retinyl-palmitate (**b)** all-trans retinol levels, and (**c)** relative hepatic mRNA transcripts of the retinoic acid receptor β2 (RARβ2) and percent steatosis (0–80%) from human livers. All mRNA levels are expressed as relative fold change compared to livers with 0% steatosis. (**C)** Representative images of double immunofluorescence stained human livers with antibodies against Crbp1 (red) and LRAT (green). Crbp1 positive SCs (yellow/orange) marked with white arrows. Magnification 200X, Scale Bars = 50 μm. (**D)** Quantitation of percentage of Crbp1 positive stellate cells (SCs) in C. 10–15 random fields per group (2–5 samples per group) were imaged and analyzed for percent SC positive Crbp1 and then pooled within tertiles of steatosis: (0–20%), (40–60%) and (70–80%).

Given that steatosis is a hallmark of obesity and often occurs without the presence of inflammation and fibrosis^37^,^38^, we sought to determine if there is also a relationship between hepatic VA levels, signaling, and steatosis in human livers that were free of liver injury. We analyzed a cohort of 33 frozen human cadaver liver samples with increasing degrees of steatosis (0–90%), but with no evidence of active steatohepatitis or fibrosis (Fig. 4A(a–d,e–h)), for levels of RP and ROL, VA signaling, and Crbp1 protein levels. With increasing steatosis hepatic levels of retinyl palmitate (RP) and retinol (ROL) decreased (Fig. 4B(a), R = −0.611, *p* < 0.001; (b), R = −0.674, p < 0.001), respectively. Consistent with these decreases in RP and ROL, there was a strong correlation between increasing steatosis and decreasing RARβ2 transcript levels, a functional measure of VA signaling (Fig. 4B(c)). Our mouse and human liver studies are compelling and strongly suggest that functional hepatic VAD in obese patients with simple steatosis (e.g. no hepatic inflammation or injury) could contribute to hepatocellular injury, leading to steatohepatitis or fibrosis.

We also detected a reduction in the percentage of Crbp1 positive VA storing SCs with increasing steatosis in these human livers (Fig. 4C,D). Our human liver data are consistent with our findings in HFD-fed and genetically obese mice and demonstrate a strong relationship among hepatic steatosis, the onset of functional tissue VAD, and reductions in SC Crbp1 expression. Thus, given the clinical correlation between obesity and steatosis in other organs, including kidney, pancreas, and lung^39^,^40^,^41^,^42^, we hypothesize that ectopic lipid accumulation, a reduction in SC Crbp1 expression, and the onset of the silent VAD phenotype in these organs are mechanistically related.

Collectively, our data show that one of the metabolic consequences of obesity is the development of an organ-specific, functional VAD that is not detectable by the current, standard clinical methods for assessing VA status, i.e., serum ROL levels. VAD resulting from malnutrition, which remains a health crisis in numerous developing countries^43^, leads to reductions in both tissue and serum VA levels^29^,^30^, evident both clinically (e.g. xerophthalmia, and increased mortality associated with infections) and non-clinically (e.g. impaired immune response, altered cell differentiation). Our data demonstrate that overnutrition and obesity, typical of western lifestyles, can also lead to major reductions in tissue VA levels. We suggest that given the essential functions of VA in humans, “silent” tissue-specific VAD has unappreciated effects on human health in obese individuals, affecting multiple organ systems.

## Methods:

### Guidelines for all Animal and Human Experimental Protocols and Methods

All experimental protocols were approved by the Institutional Animal Care and Use Committee (IACUC) at Weill Cornell Medical College in accordance with all applicable federal, state and local regulations. All methods were carried out in accordance with approved guidelines. *Human Liver Studies:* Studies using human liver tissue obtained from the Liver Tissue Cell Distribution System (LTCDS), (Minneapolis, Minnesota) do not constitute “Human Subjects Research” and do not require review by the Weill Cornell Medical College Institutional Review Board (IRB) as per OHRP regulations (45CFR46.102).

### Animals & Diets

#### High fat diet induced obesity studies

7–8 week old wt C57BL/6 male mice were fed either a standard laboratory chow-fed diet (Con, n = 5) with 13% kcal from fat (diet# 5053, 15 IU of vitamin A-acetate/gram, Pico Rodent Diet, Lab-Diets Co, St. Louis, MO), a commercial control diet (Con, n = 4) with 10% kcal from fat (diet #58124, 3.8 IU vitamin A-acetate/gram, Test-Diets Co, St. Louis, MO) or a high fat diet (HFD, n = 5) with 45% kcal from fat, (diet #58125, 4.7 IU vitamin A-acetate/gram, Test-Diets Co, St. Louis, MO) for 4 months. We conducted experiments and determined that HFD-fed wt mice had significant reductions of tissue VA when compared to wt mice fed either the commercial control diet (Test-Diet, #58124, 10% kcal fat, 3.8 IU vitamin A-acetate/gram) or the standard laboratory chow diet used in the Weill Cornell Medical College (WCMC) Vivarium (Pico Rodent Diet, #5053, 13% fat, 15 IU/vitamin A-acetate). Mice were sacrificed and tissue and serum samples collected and stored protected from light at −70 °C until further analysis. Experiments were performed 3–5 times with different cohorts of mice.

#### Obesity reversal studies

Cohorts of obese wt C57BL/6 mice previously fed a HFD for 4 months were switched back to a chow diet for 6 weeks. After 6 weeks, mice were sacrificed and tissue and serum samples collected and stored protected from light at −70 °C until further analysis.

#### Genetic models of obesity

7–8 week old *ob/ob* (n = 3) and *db/db* (n = 3) mice, which spontaneously develop obesity by 9–10 weeks of age^25^,^26^, were fed a standard lab diet (13% kcal fat, Pico Rodent Diet #5053, Lab-Diets Co, St. Louis, MO,) *ad libitum* for 4 weeks. After 4 weeks, *ob/ob*, *db/db* mice, and age-matched chow-fed wt C57BL/6 control mice (n = 3) were sacrificed and tissue and serum samples collected and stored protected from light at −70 °C until further analysis.

### High Performance Liquid Chromatography (HPLC) of Tissue Vitamin A

Tissue vitamin A was extracted and analyzed by HPLC as previously described^44^. Retinol and retinyl-palmitate were identified by HPLC based on two criteria: an exact match of the retention times of peaks with those of authentic retinoid standards detected at a wavelength of 325 nm, and identical ultraviolet light spectra (220–400 nm) of unknowns against spectra from authentic retinoid standards during HPLC by the use of a photodiode array detector. Tissue vitamin A levels were normalized to mg of wet tissue weight or volume for serum.

### Serum Retinol Binding Protein-4 (RBP4) Measurements

Serum RBP4 was measured using the Dual Mouse/Rat RBP4 ELISA kit from AdipoGen, Corp (San Diego CA) according to the manufacturer’s protocol.

### RNA Isolation, cDNA, and Quantitative RT-PCR (Q-RT-PCR)

Total RNA was isolated from mouse and human tissues using RNeasy mini kits (Qiagen, Valencia, CA) and quantified using a Nano Drop 2000 spectrophotometer (Thermo Scientific, Wilmington, DE). Total RNA (2 μg) was used to synthesize cDNA with random primers using a qScript cDNA synthesis kit (Quanta Biosciences, Gaithersburg, MD). Q-RT-PCR was performed using a SYBR Green PCR master mix on a Bio-Rad MyiQ2 Real Time PCR iCycler (Bio-Rad, Inc. Hercules, CA). Gene specific primers (Table S1) were used to amplify mRNA target genes. cDNA from 3–9 mice and 3–4 human samples per experimental group were analyzed for relative mRNA fold changes, calculated using the delta CT method, which were normalized to an Hprt internal control gene.

### Immunofluorescence Microscopy

Paraffin embedded or frozen mouse and human tissue sections were incubated with antibodies against: Crbp1 (RBP1) (mouse monoclonal, 1:300, sc-271208, Santa Cruz Inc, Santa Cruz, CA) LRAT (rabbit polyclonal, 1:300, # sc-99016 Santa Cruz Inc, Santa Cruz, CA), Vimentin (goat polyclonal 1:500, #sc-7558, Santa Cruz Inc., Santa Cruz, CA) at 4 °C overnight. We used Alexa-fluor 594 and 488 conjugated anti-rabbit (1:500), anti-mouse (1:500) anti-goat (1:500) secondary antibodies (Invitrogen, Carlsbad, CA) for immunofluorescence labeling of targets followed by visualization using a Nikon TE2000 inverted fluorescence microscope (Nikon, Inc). The average percentage positive LRAT/Crbp1+ or Vimentin/Crbp1+ stellate cells was determined as previously described^45^. The background fluorescence levels were averaged from 10 random fields and used to determine a minimum threshold for positive staining for green channel and red channel, which was maintained for all images analyzed in each experimental group. Positive LRAT/Crbp1+ or Vimentin/Crbp1+ was calculated by determining the number of pixels shared by green and red channels in each field divided by the total number of green and red positive pixels within each field analyzed. 10–15 random fields per mouse section, with 3–4 sections per mouse, 3 or >mice/group were imaged and analyzed using Nikon NIS elements AR advanced imaging software suite (Nikon, Inc).

### Liver Histology

To determine liver histology and steatosis, mouse and human livers were fixed in 4% formaldehyde buffer and embedded in paraffin. Paraffin-embedded liver sections were cut, mounted on glass slides, stained with hematoxylin and eosin, and scored by Dr. Jose Jessurun, using established criteria, in a blinded manner^46^. To determine liver collagen deposition and fibrosis, mouse and human liver sections were stained with Masson’s Trichrome Kit (Poly Scientific, Bayshore, NY), according to the manufacturers’ protocol.

### Human Liver Studies

Frozen human livers were obtained from the Liver Tissue Cell Distribution System (LTCDS), (Minneapolis, Minnesota), which was funded by NIH Contract # HHSN276201200017C. We selected a cohort of 37 frozen human livers with the following criteria: Sex: Male, Average age: 36 (±1.36) years, Causes of death: (i) motor vehicle accident or (ii) head trauma. Due to the circumstances of LTCDS acquisition of human donor liver samples, anthropometric data (height, weight) of human liver donors were unavailable and therefore we were unable to determine body mass index of the liver donors. Frozen human livers were fixed in 4% paraformaldehyde and embedded in paraffin wax. Liver samples underwent a complete histopathology evaluation by the Department of Surgical Pathology at New York-Presbyterian Hospital/WCMC and were scored for evidence of steatohepatitis, fibrosis and percent (%) fatty liver (steatosis). Three liver samples with evidence of steatohepatitis and fibrosis where removed from our cohort and a total of 33 human livers with a range of steatosis (0–90%) and no evidence of steatohepatitis or fibrosis were selected and analyzed for vitamin A content by HPLC, mRNA levels by qRT-PCR, and protein expression by immunofluorescence microscopy.

### Statistics

HPLC analysis, q-RT-PCR for relative mRNA levels, histology and immunofluorescence quantitation values are reported as mean ± standard error of the mean (±SEM) with 3–9 mice per group. Significant differences, defined as p-values less than an alpha of 0.05, were calculated using one-way analysis of variance followed by Bonferroni multiple comparison post-hoc analysis. Errors bars with *p < 0.05, **p < 0.01, ***p < 0.001, ****p < 0.0001 are relative to chow-fed wt mice (blue bars). For time course experiments (Fig. 3), significances differences in hepatic VA and mRNA levels are relative to the means of chow-fed wt mice (blue bars), which include all controls from 1M, 2M, and 4M cohorts. Linear relationships between percent steatosis, VA levels, and relative mRNA levels in human livers were determined by standard least-squares regression analysis. For all statistical analyses outliers were pre-defined as any value that is more than 2 standard deviations from the mean. All statistical analyses were performed using GraphPad Prism 6.0 statistical software (GraphPad Software, San Diego, CA).

## Additional Information

**How to cite this article**: Trasino, S. E. *et al.* Obesity Leads to Tissue, but not Serum Vitamin A Deficiency. *Sci. Rep.* **5**, 15893; doi: 10.1038/srep15893 (2015).

## Supplementary Material

Supplementary Dataset
