# Supplementary material for: Obesity Leads to Tissue, but not Serum Vitamin A Deficiency
Source: Sci Rep. 2015 Nov 2;5:15893. doi: 10.1038/srep15893 (PMC4629132; doi:10.1038/srep15893)
Supplement: Supplementary Dataset [file srep15893-s1.doc]

Obesity Leads to Tissue, but not Serum Vitamin A Deficiency

Steven E. Trasino^1^, Xiao-Han Tang,^1^ Jose Jessurun^2^, Lorraine J. Gudas^1*^

Affiliations: ^1^Department of Pharmacology, ^2^Department of Pathology,

Weill Cornell Medical College of Cornell University, New York, NY 10065

^*^To whom correspondence should be addressed: Lorraine J. Gudas, Department of Pharmacology,

Weill Cornell Medical College of Cornell University, 1300 York Ave., New York, NY 10065.

Tel.: 212-746-6250; Fax: 212-746-8858; E-mail: ljgudas@med.cornell.edu.

**Supplemental Data Figure Legends:**

**Fig. S1. Chromatographic tracings of Vitamin A.** Chromatographic tracings of Vitamin A extracted from livers of (**A)** (wt) C57/BL6 mice fed either a standard lab chow diet (chow), **(B)** high fat diet (HFD) for 4 months, or 12 week-old **(C)** *ob/ob* mice and **(D)** *db/db* mice fed a standard lab chow diet for 4 weeks. All-trans retinol (ROL-black arrow, ~32 minute retention time) and retinyl-palmitate (RP-black arrow, ~55 minute retention time) were detected at a wavelength of 325 nm and identified by a match of retention times of pure retinoid standards and identical ultraviolet light spectra (220–400 nm) of unknowns against spectra from authentic retinoid standards during HPLC by the use of a photodiode array detector. We measured extraction efficiency and normalized data to account for differences in extraction efficiency by using retinyl acetate (RAc-black arrow), which, as an internal standard, eluted at ~37.3 minutes. Two retinoids with retention times of ~51 and ~53 minutes respectively (* red asterisk, (**A)**), were identified in all liver samples **(A-D).** Using the same retinoid extraction and HPLC protocols used in this project, previously published work in our laboratory concluded that the unidentified retinoids with elution times of ~51 and ~53 minutes are not taken up by murine cells in culture ^1^, and therefore were excluded from our current analysis.

**Fig. S2. Tissue mRNA Transcript Levels of Vitamin A Regulated Genes.** Relative mRNA transcript levels of retinoic acid receptors **α** and γ (RARα, RARγ in **(A-B)** liver, **(C-D)** pancreas, **(E-F)** kidney and **(G-H)** lung from (wt) C57/BL6 mice fed either a standard lab chow diet (chow) or high fat diet (HFD) for 4 months, and 12 week-old *ob/ob* mice and *db/db* mice fed a standard lab chow diet for 4 weeks. ND=not detected. Errors bars represent ± SEM of 3-4 mice per group.

**Fig. S3. Obese mice with Decreased Liver Vitamin A Show No Evidence of Liver Injury at 4 Months. (A)** Representative images of trichrome-stained livers from wild type (wt) C57/BL6 mice fed either a standard lab chow diet (chow) or high fat diet (HFD) for 4 months. Magnification 200X, Scale Bars =50 μm. **(B)** Representative images of hematoxylin and eosin stained livers from wild type (wt) C57/BL6 mice fed either a standard lab chow diet (chow) or high fat diet (HFD) for 4 months, and wt mice that were previously obese and then switched back to a chow diet for 6 weeks (HFDR). Magnification 200X, Scale Bars =50 μm.

**Table 1.** Primer sequences for Quantitative Real Time Gene Expression Analysis.

**Supplemental References:**

1 Guo, X., Morris, P. & Gudas, L. Follicle-stimulating hormone and leukemia inhibitory factor regulate Sertoli cell retinol metabolism*. Endocrinolo*g**y 1**42, 1024-1032, doi:10.1210/endo.142.3.7996 (2001).

**Supplemental Figures:**
